# Reasons for acute referrals to hospital from general practitioners and out-of-hours doctors in Norway: a registry-based observational study

**DOI:** 10.1186/s12913-021-07444-7

**Published:** 2022-01-15

**Authors:** Jesper Blinkenberg, Øystein Hetlevik, Hogne Sandvik, Valborg Baste, Steinar Hunskaar

**Affiliations:** 1 National Centre for Emergency Primary Health Care, NORCE Norwegian Research Centre AS, Årstadveien 17, 5009 Bergen, Norway; 2 Department of Global Public Health and Primary Care, University of Bergen, Årstadveien 17, 5009 Bergen, Norway

**Keywords:** Norway, General practitioners, After-hours care, Out-of-hours medical care, Gatekeeping, Referral and consultation, Emergencies, Patient admission, ICPC-2, Abdominal pain, Chest pain, Shortness of breath, Pneumonia, Appendicitis, Myocardial infarction, Stroke

## Abstract

**Background:**

General practitioners (GPs) and out-of-hours (OOH) doctors are gatekeepers to acute hospital admissions in many healthcare systems. The aim of the present study was to investigate the whole range of reasons for acute referrals to somatic hospitals from GPs and OOH doctors and referral rates for the most common reasons. We wanted to explore the relationship between some common referral diagnoses and the discharge diagnosis, and associations with patient’s gender, age, and GP or OOH doctor referral.

**Methods:**

A registry-based study was performed by linking national data from primary care in the physicians’ claims database with hospital services data in the Norwegian Patient Registry (NPR). The referring GP or OOH doctor was defined as the physician who had sent a claim for the patient within 24 h prior to an acute hospital stay. The reason for referral was defined as the ICPC-2 diagnosis used in the claim; the discharge diagnoses (ICD-10) came from NPR.

**Results:**

Of all 265,518 acute hospital referrals from GPs or OOH doctors in 2017, GPs accounted for 43% and OOH doctors 57%. The overall referral rate per contact was 0.01 from GPs and 0.11 from OOH doctors, with large variations by referral diagnosis. Abdominal pain (D01) (8%) and chest pain (A11) (5%) were the most frequent referral diagnoses. For abdominal pain and chest pain referrals the most frequent discharge diagnosis was the corresponding ICD-10 symptom diagnosis, whereas for pneumonia-, appendicitis-, acute myocardial infarction- and stroke referrals the corresponding disease diagnosis was most frequent. Women referred with chest pain were less likely to be discharged with ischemic heart disease than men.

**Conclusions:**

The reasons for acute referral to somatic hospitals from GPs and OOH doctors comprise a wide range of reasons, and the referral rates vary according to the severity of the condition and the different nature between GP and OOH services. Referral rates for OOH contacts were much higher than for GP contacts. Patient age, gender and referring service influence the relationship between referral and discharge diagnosis.

## Background

For patients with acute conditions visiting a general practitioner (GP) or an out-of-hours (OOH) doctor, referral to acute admission to hospital is sometimes required in order to obtain adequate investigation and treatment. These acute admissions to hospitals constitute a large part of hospital activity and in the years 2015–2019, more than two thirds of all admissions in Norway were acute [1]. Patients referred acute to hospital comprise all age groups, but the elderly have a higher incidence of acute severe disease and are more frequently admitted to hospital [2, 3]. Health authorities worldwide are concerned about emergency department overcrowding, and that extended diagnostic possibilities and a development towards more defensive medicine will put the health services under considerable stress [4–6].

A gatekeeping system, where the patients are obliged to see a GP or an OOH doctor before referral, may regulate the access to acute hospital care, and is implemented in many healthcare systems. Gatekeeping may reduce unplanned hospital admissions in general, and especially for the elderly [7, 8]. In the Norwegian healthcare system, the GPs and OOH doctors are gatekeepers to specialist care, including hospital admissions, for all kind of emergencies, including traumas. In a previous study we found that two thirds of acute admissions to hospitals in Norway 2014 came after contact with a GP or an OOH-doctor [9, 10], the rest were direct admissions of different kinds.

Discharge diagnoses after acute admissions in Norway have been well described, and similar diagnoses for patients admitted to hospital after emergency ambulance transport in Denmark are published [9–11]. However, reasons for acute referrals to hospital from GPs and OOH doctors specifically, and potentially different patterns and rates between these referral agents have not been explored in detail.

Abdominal pain and chest pain are known as two dominant clinical symptom groups in OOH services and acute referrals to hospital from primary care [12, 13]. Likewise, pneumonia, appendicitis, acute myocardial infarction (AMI) and stroke are important referral diagnoses in terms of both numbers and severity [14–17]. Nevertheless, the referral rates for these clinical presentations and conditions have not been thoroughly investigated. Furthermore, there is little knowledge of the relationship between the referral diagnosis from GP and OOH doctors and the subsequent discharge diagnosis from hospital.

The aim of the present study was therefore to investigate the whole range of reasons for acute referral to somatic hospital stay from GPs and OOH doctors in Norway, including referral rates for the most frequent ICPC-2 diagnoses. For three common clinical problems, abdominal pain, chest pain, and shortness of breath, and for the frequent or important referral diagnoses of pneumonia (R81), appendicitis (D88), AMI (K75), and stroke (K90), we also wanted to investigate the associations between the diagnosis given in referral contacts and the discharge diagnosis from hospital, and how these associations varied between GPs and OOH doctors and with patient’s gender and age.

## Methods

Norway has a well-established public primary healthcare system including a Regular General Practitioners scheme (RGPs) and OOH services [9, 10]. RGPs provide healthcare for both acute and non-acute cases including follow up, whereas the OOH services provide care in acute cases outside the opening hours of RGPs’ surgeries. The municipalities are responsible for the primary healthcare, including RGPs and OOH services. The state organizes the specialist care, including ambulances and hospitals.

### Data sources

The study is registry-based, using data from national health registries covering the whole population in Norway in 2017.

All GP and OOH contacts result in a claim to the Control and Payment of Reimbursement to Health Service Providers database (KUHR), managed by The Norwegian Health Economics Administration (HELFO). A single claim contains the patient’s national identification number, time and date for the contact, and if the contact is a simple contact (e.g. telephone contact), an office consultation, or a home visit. Also, it is mandatory to include one or more diagnostic codes according to International Classification of Primary Care (ICPC-2) [18]. This diagnosis will be routinely transferred to the referral letter and recorded as the reason for referral. In our material, 17% of the KUHR claims contained more than one diagnosis. We used the main (primary) diagnosis in our analyses. The ICPC-2 codes are divided into chapters denoted by a letter that identifies an organ system, followed by a two-digit number referring to either a symptom (code 01–29) or a disease (code 70–99). ICPC-2 was developed to describe the reasons for encounter in primary health care or general practice and reflects the content of primary care, last updated in 2016 [19, 20].

Psychiatric hospitals were not included. We therefore use the term somatic hospital admissions in our study. All hospital stays are recorded in the Norwegian Patient Registry (NPR), which includes information on patient’s national identification number, time and date of the admission, degree of urgency, and one or more discharge diagnoses using the International Statistical Classification of Diseases and Related Health Problems version 10 (ICD-10). For the hospital discharge diagnoses we used the main diagnosis with three characters. An acute hospital admission was defined by the NPR’s data form as an admission required immediately or within 24 h and lasting for more than 24 h. These admissions were then included if they were related to a GP or OOH contact.

### Study population, variables and definitions, and linkage procedures

In this study we defined a GP or OOH doctor contact as a consultation or a home visit, telephone contacts were excluded. All such contacts with GPs (*N* = 14,457,247) or OOH doctors (*N* = 1,361,731) during 2017 were included. For GPs this represent both acute and follow up contacts.

Statistics Norway (SSB) created a pseudo-anonymized identification number which replaced the national identification number in the KUHR and NPR databases. Thereby data from both sources could be combined without revealing the patients’ identities.

A GP or OOH-doctor contact in KUHR was defined as the referral contact for a patient if it occurred within 24 h before an acute admission in NPR. By this definition we identified 265,518 referrals to hospital from a GP or an OOH doctor.

A suitable grouping of ICPC-2 codes into presenting complaints in emergency departments (EDs) has been described by Malmström et al. [21]. We applied this for the symptom groups abdominal pain, chest pain and shortness of breath. The abdominal pain symptom group was defined by the following codes: abdominal pain/cramps general (D01), abdominal pain epigastric (D02) and abdominal pain localized other (D06). The chest pain symptom group was defined by these codes: chest pain NOS (A11), heart pain (K01), pressure/tightness of heart (K02) and cardiovascular pain NOS (K03). Shortness of breath was included pain in respiratory system (R01), shortness of breath/dyspnoea (R02), wheezing (R03), and breathing problem other (R04). This group was named after the most frequent reason, shortness of breath.

We used the referral disease diagnoses pneumonia (R81), appendicitis (D88), acute myocardial infarction (K75), and stroke (K90) to study associations between a specific disease diagnosis given at referral and the discharge diagnoses after hospital stay.

### Statistical analyses

Numbers and frequencies for ICPC-2 chapters and the 30 most common ICPC-2 diagnoses that led to a referral were obtained. Referral rates were calculated by dividing GP and OOH contacts leading to a referral by all contacts with the same diagnosis.

For referrals in the abdominal pain symptom group, we identified the ten most frequent discharge diagnoses. Generalized linear model (GLM) log-binomial regression were used to estimate relative risk (RR) of the patient being discharged with each of these diagnoses compared with all other discharge diagnoses after a referral with abdominal pain symptom. The patients’ ages, genders and if the patient had been referred by a GP or OOH doctor were used as explanatory variables and were adjusted for each other. RR was calculated for females with males as reference, age was divided by 10 and then used as a continuous variable giving RR with a 10-years increase, and OOH doctor referrals were compared with GP referrals as reference. The RRs were presented with 95% confidence intervals (CI). For referrals in both the chest pain symptom group and shortness of breath symptom group the ten most frequent discharge diagnoses were identified, and equivalent GLM were used for each of the ten discharge diagnoses.

For the four disease-specific referral diagnoses pneumonia (R81), appendicitis (D88), AMI (K75) and stroke (K90) we conducted frequency analyses for discharge ICD-10 diagnoses representing > 3% of the patients referred with these diagnoses, respectively. GLM were used to estimate the RR for the discharge diagnoses. For the referral diagnosis pneumonia (R81) the corresponding discharge ICD-10 diagnosis pneumonia was used as comparison. For appendicitis (D88), AMI (K75) and stroke (K90) we used respectively acute appendicitis (K35), AMI (I21) and cerebral infarction (I63) as comparison in the analyses. Explanatory variables were gender, age, and referral from an OOH doctor or a GP. For three of the models the log-binomial regression did not converge, and a Poisson regression with robust variance estimates was applied to estimate RR [22].

The analyses were performed using Stata 16.1. (StataCorp. 2019. Stata Statistical Software: Release 16. College Station, TX: StataCorp LLC.)

## Results

In 2017 GP and OOH-doctor contacts (consultations and home visits) resulted in 265,518 referrals to acute somatic hospital admission, 150,577 after OOH contacts and 114,941 after GP contacts (Table 1). The mean patient age for referrals was 56 years, whereas for all contacts the mean age was 47 years. Women accounted for 50% of the patients referred, but 58% of all the contacts.

**Table 1:** ICPC-2 diagnosis chapter for GP and OOH contacts leading to acute referrals to hospitals

	Referrals		GP referrals		OOH referrals	
	N	% of total	N	% of total referrals	N	% of total referrals
**Total**	**265,518**	**100**	**114,941**	**43**	**150,577**	**57**
**ICPC-2 diagnosis chapter**^a^						
General and unspecified (A)	47,821	18	16,067	6	31,754	12
Blood and immune mechanisms (B)	3539	1	2574	1	965	0
Digestive (D)	49,666	19	20,454	8	29,212	11
Eye (F)	2172	1	1063	0	1109	0
Ear (H)	890	0	471	0	419	0
Cardiovascular (K)	38,927	15	19,989	8	18,938	7
Musculoskeletal (L)	26,524	10	11,004	4	15,520	6
Neurological (N)	20,287	8	7858	3	12,429	5
Psychological (P)	8707	3	2809	1	5898	2
Respiratory (R)	34,936	13	16,174	6	18,762	7
Skin (S)	8255	3	3897	1	4358	2
Endocrine and metabolic (T)	5702	2	3411	1	2291	1
Urological (U)	10,432	4	4667	2	5765	2
Pregnancy and family planning (W)	3993	2	2588	1	1405	1
Female genital (X)	1415	1	814	0	601	0
Male genital (Y)	1960	1	1029	0	931	0
Social problems (Z)	227	0	31	0	196	0

Legend: Distribution of ICPC-2 diagnosis chapter for GP and OOH consultations and home visits leading to referral to acute admissions to hospitals in Norway 2017

^a^Process codes counted for 65 emergency referrals, 41 by GP and 24 by OOH doctors

GP and OOH contacts leading to referral had a similar distribution of ICPC-2 chapters. General and unspecified (A), digestive (D) and cardiovascular (K) were the most frequent chapters used (Table 1). OOH referrals had a higher share of the chapter general and unspecified (A), whereas GP referrals had a higher share of cardiovascular (K).

The 30 most common referral diagnoses accounted for 53% of the referrals, with abdominal pain/cramps general (D01) (8%), chest pain (A11) (5%) and pneumonia (R81) (3%) being the most frequent (Table 2). Of all 14,457,247 GP contacts, the referral rate was only 0.01, whereas the rate for OOH doctor contacts was 0.11 of all 1,361,731 contacts. The diagnosis with the highest referral rate both from GPs and OOH doctors was appendicitis (D88), with 0.30 and 0.79, respectively. Patients referred from OOH doctors were younger than patients referred from the GPs. The lowest patient median age was for OOH patients referred with acute bronchitis/bronchiolitis (R78) (2 years and interquartile range (IQR) 1–69) and appendicitis (D88) (26 years, IQR 18–39). The diagnosis with the oldest patients was heart failure (K77) with median age of 82 years for GP referrals and 83 years for OOH referrals with IQR 74–88 and 74–89 respectively.

**Table 2:** Frequency of ICPC-2 diagnoses by GP and OOH doctor when referring to acute hospital admission

Referral diagnoses ICPC-2	All referrals (265,518)		GP				OOH			
				Referrals (114,941)				Referrals (150,577)		
	N	%	Contacts^a^ N	Referral rate	Age Median (IQR)	% Female	Contacts^a^ N	Referral rate	Age Median (IQR)	% Female
**Total**	**265,518**	**100**	**14,457,247**	**0.01**	**64 (43–77)**	**51**	**1,361,731**	**0.11**	**59 (34–75)**	**50**
Abdominal pain/craps general (D01)	21,260	8	204,152	0.04	48 (28–67)	61	52,550	0.26	45 (27–65)	59
Chest pain NOS (A11)	14,077	5	45,280	0.10	64 (52–75)	42	26,088	0.37	62 (50–74)	43
Pneumonia (R81)	8793	3	83,218	0.05	72 (59–82)	52	22,181	0.21	75 (61–84)	48
Shortness of breath/dyspnoea (R02)	7806	3	63,345	0.05	71 (57–82)	47	14,029	0.32	71 (49–82)	50
Atrial fibrillation/flutter (K78)	6892	3	290,943	0.01	74 (67–82)	43	6323	0.45	71 (61–79)	46
COPD (R95)	5643	2	105,601	0.03	74 (68–80)	52	10,852	0.28	73 (67–80)	54
Heart pain (K01)	5319	2	8976	0.20	64 (53–74)	46	5310	0.66	62 (50–73)	45
Vertigo/dizziness (N17)	5082	2	99,867	0.02	69 (52–79)	60	12,499	0.22	70 (53–80)	54
Fainting/ syncope (A06)	4353	2	19,402	0.07	70 (47–81)	51	9356	0.31	68 (44–79)	50
Infectious disease other (A78)	4162	2	32,656	0.04	66 (44–77)	47	7773	0.35	70 (49–81)	44
Stroke/cerebrovascular disease (K90)	4078	2	41,866	0.04	74 (65–83)	48	2986	0.78	73 (62–82)	47
Fever (A03)	3895	1	42,768	0.03	34 (2–66)	48	17,854	0.14	32 (1–69)	44
Abdominal pain localized other (D06)	3812	1	43,181	0.04	46 (28–66)	64	9259	0.24	39 (25–60)	62
Headache (N01)	3624	1	108,973	0.01	43 (29–61)	60	12,972	0.17	42 (28–61)	62
Disease/condition unspecified (A99)	3544	1	129,088	0.01	60 (38–75)	50	33,417	0.07	56 (31–75)	50
Heart failure (K77)	3069	1	54,594	0.04	82 (74–88)	42	2543	0.43	83 (74–89)	46
Ischaemic heart disease with angina (K74)	2995	1	24,769	0.07	70 (60–79)	36	2042	0.65	69 (58–79)	41
Skin infection other (S76)	2682	1	37,180	0.03	63 (48–74)	42	12,341	0.12	60 (44–73)	40
Cystitis/urinary infection other (U71)	2632	1	182,861	0.01	72 (57–82)	55	50,608	0.03	73 (55–84)	50
Hip symptom/complaint (L13)	2614	1	79,674	0.01	78 (67–87)	67	6185	0.27	81 (70–87)	65
Injury musculoskeletal system NOS (L81)	2595	1	67,921	0.01	64 (38–78)	46	24,595	0.07	57 (27–76)	47
Appendicitis (D88)	2581	1	3377	0.30	30 (18–47)	50	1990	0.79	26 (18–39)	55
Transient cerebral ischaemia (K89)	2536	1	9263	0.12	72 (61–81)	55	1982	0.73	73 (63–82)	52
Concussion (N79)	2465	1	18,661	0.03	25 (9–64)	46	9489	0.20	29 (13–64)	43
Pyelonephritis/pyelitis (U70)	2353	1	9624	0.09	59 (36–73)	65	5399	0.27	56 (30–74)	66
Disease digestive system other (D99)	2293	1	24,280	0.04	64 (50–76)	51	2533	0.57	63 (48–75)	47
Cholecystitis / cholelithiasis (D98)	2249	1	19,713	0.05	59 (44–72)	58	5847	0.21	52 (37–66)	62
Acute bronchitis/bronchiolitis (R78)	2147	1	138,933	0.01	29 (1–70)	48	19,238	0.05	2 (1–69)	44
Palpitations /awareness of heart (K04)	2083	1	42,869	0.02	66 (50–78)	58	8706	0.15	63 (44–75)	56
Abdominal pain epigastric (D02)	1980	1	39,268	0.02	54 (35–70)	61	7198	0.18	51 (34–67)	61
Sum 30	139,614	53								

Legend: The frequency of the 30 most common ICPC-2 diagnoses used by general practitioners (GPs) and out-of-hours (OOH) doctors when referring to acute admission to hospitals in Norway 2017, presented for all admissions, and for GP daytime practice and OOH services separately

*IQR* Interquratile range

^a^Contact is defined as a consultation or home visit

Referrals with an ICPC-2 diagnosis in the symptom groups abdominal pain (27,052 patients), chest pain (19,546 patients) or shortness of breath (8371 patients) accounted for 21% of all referrals (Tables 3, 4, 5).

**Table 3:** Discharge diagnoses for patients referred to acute hospital admission with abdominal pain (D01, D02 and D06)

Discharge ICD-10 diagnoses	All		OOH referrals			Age			Gender (F)		
	N	%	%^a^	RR^c^	95% CI	Median (IQR)	RR^c^	95% CI	%^b^	RR^c^	95% CI
Abdominal and pelvic pain (R10)	7127	26	64	1.02	0.98–1.06	34 (23–52)	0.86	0.85–0.86	68	1.38	1.32–1.44
Acute appendicitis (K35)	3166	12	63	0.98	0.92–1.05	32 (20–50)	0.80	0.79–0.81	48	0.59	0.55–0.62
Cholelithiasis (K80)	1664	6	66	1.17	1.06–1.28	58 (42–73)	1.21	1.19–1.24	56	0.89	0.81–0.98
Diverticular disease (K57)	1483	5	45	0.51	0.46–0.56	61 (51–72)	1.31	1.28–1.34	60	1.09	0.99–1.20
Functional intestinal disorder (K59)	1141	4	64	1.06	0.94–1.19	56 (28–76)	1.11	1.09–1.14	58	0.94	0.84–1.06
Ileus (K56)	1031	4	78	2.12	1.84–2.45	65 (50–76)	1.34	1.30–1.38	52	0.81	0.72–0.92
Acute pancreatitis (K85)	814	3	69	1.32	1.14–1.52	57 (44–71)	1.18	1.15–1.22	43	0.54	0.47–0.61
Calculus of kidney and ureter (N20)	666	2	65	1.07	0.91–1.25	51 (36–66)	1.07	1.04–1.11	37	0.40	0.34–0.47
Noninfl. disorders of ovary. f. tube. broad lig (N83)	448	2	67	1.20	0.99–1.45	31 (23–40)	0.75	0.72–0.78	100	1	
Other gastroenteritis and colitis (A09)	417	2	59	0.81	0.67–0.98	33 (21–52)	0.83	0.80–0.87	64	1.10	0.90–1.34
Other	9095	34	62			50 (30–71)			61		
All	27,052	100	63			46 (27–66)			60		

Legend: Distribution of discharge diagnoses for patients referred to acute hospital admission by general practitioner (GP) and out-of-hours (OOH) doctor with the ICPC-2 diagnosis: abdominal pain (D01, D02 and D06) in Norway 2017

*IQR* Interquartile range

^a^Percent of referrals with abdominal pain-diagnosis (D01, D03 or D06) and the current ICD-10 discharge diagnosis which are referred by OOH doctor

^b^Percent of women in referrals with abdominal pain-diagnosis (D01, D03 or D06) and the current ICD-10 discharge diagnosis

^c^Relative risk for the different discharge ICD-10 diagnoses for OOH referrals relative to GP referrals, for a 10-years increase in age, and for female patients relative to male patients* compared with all discharge diagnoses after abdominal pain admission*

**Table 4:** Discharge diagnoses for patients referred to acute hospital admission with chest pain (A11, K01, K02 and K03)

Discharge ICD-10 diagnoses	All		OOH referrals			Age			Gender (F)		
	N	%	%^a^	RR^c^	95% CI	Median (IQR)	RR^c^	95% CI	%^b^	RR^c^	95% CI
Pain in throat and chest (R07)	6965	36	69	0.98	0.95–1.02	56 (46–68)	0.85	0.84–0.86	46	1.15	1.11–1.19
Acute myocardial infarction (I21)	2277	12	66	0.98	0.90–1.06	68 (59–77)	1.30	1.26–1.33	32	0.54	0.50–0.59
Angina pectoris (I20)	1873	10	61	0.77	0.71–0.84	69 (58–78)	1.27	1.24–1.31	38	0.70	0.64–0.77
Other soft tissue disorder incl. myalgia (M79)	992	5	68	0.95	0.83–1.08	56 (45–68)	0.82	0.79–0.85	51	1.48	1.31–1.68
Chronic ischaemic heart disease (I25)	758	4	68	1.05	0.90–1.22	65 (56–74)	1.17	1.12–1.22	28	0.47	0.40–0.55
Atrial fibrillation and flutter (I48)	573	3	72	1.27	1.06–1.51	73 (66–82)	1.59	1.50–1.69	46	0.88	0.75–1.04
Pneumonia (J12–18)	385	2	72	1.26	1,01-1,57	70 (59–81)	1.30	1.21–1.38	42	0.80	0.66–0.98
Heart failure (I50)	250	1	69	1.13	0.87–1.48	80 (70–88)	2.13	1.93–2.36	43	0.67	0.52–0.87
Essential (primary) hypertension (I10)	239	1	63	0.82	0.63–1.06	67 (57–75)	1.13	1.04–1.22	58	1.70	1.31–2.19
Gastro-oesophageal reflux disease(K21)	237	1	64	0.83	0.63–1.07	62 (51–74)	1.01	0.93–1.09	50	1.27	0.99–1.65
Other	5125	26	70			64 (50–76)			47		
All	19,546	100	68			62 (50–74)			44		

Legend: Distribution of discharge diagnoses for patients referred to acute hospital admission by general practitioner (GP) and out-of-hours (OOH) doctor with the ICPC-2 diagnosis: chest pain (A11, K01, K02 and K03) in Norway 2017

*IQR* Interquartile range

^a^Percent of referrals with chest pain-diagnosis (A11, K01, K02 and K03) and the current ICD-10 discharge diagnosis which are referred by OOH doctor

^b^Percent of women in referrals with chest pain-diagnosis (A11, K01, K02 and K03) and the current ICD-10 discharge diagnosis

^c^Relative risk for the different discharge ICD-10 diagnoses for OOH referrals relative to GP referrals, for a 10-years increase in age, and for female patients relative to male patients *compared with all discharge diagnoses after chest  pain admission*

**Table 5:** Discharge diagnoses for patients referred to acute hospital admission with shortness of breath (R01, R02, R03 and R04)

Discharge ICD-10 diagnoses	All		OOH referrals			Age			Gender (F)		
	N	%	%^a^	RR^c^	95% CI	Median (IQR)	RR^c^	95% CI	%^b^	RR^c^	95% CI
Heart failure (I50)	991	12	57	1,00	0,89-1,12	82 (73–88)	1,60	1,53-1,68	41	0,66	0,59-0,74
Pneumonia (J12–18)	943	11	67	1,48	1,30-1,68	73 (59–83)	1,08	1,05-1,11	50	1,04	0,92-1,17
Chronic obstructive pulmonary disease (J44)	633	8	62	1,2	1,03-1,40	74 (67–82)	1,25	1,20-1,30	55	1,19	1,03-1,39
Abnormalities of breathing (R06)	529	6	50	0,66	0,56-0,78	62 (38–76)	0,92	0,90-0,95	56	1,39	1,17-1,64
Pulmonary embolism (I26)	371	4	49	0,69	0,56-0,84	66 (52–76)	1,02	0,98-1,06	45	0,87	0,71-1,06
Atrial fibrillation and flutter (I48)	298	4	41	0,52	0,42-0,65	76 (69–84)	1,36	1,26-1,45	43	0,76	0,61-0,96
Asthma (J45)	288	3	68	1,20	0,94-1,54	31 (3–60)	0,75	0,72-0,77	50	1,10	0,88-1,38
Pain in throat and chest (R07)	277	3	55	0,80	0,64-1,02	53 (38–68)	0,9	0,87-0,94	58	1,49	1,18-1,89
Acute bronchiolitis (J21)^d^	213	3	75	1,17	0,89-1,54	1 (1–1)			39	0,92	0,72-1,17
Other acute lower respiratory infection (J22)	175	2	57	0,82	0,61-1,11	59 (13–76)	0,86	0,82-0,90	55	1,38	1,02-1,85
Other	3653	44	59			69 (47–81)			48		
All	8371	100	59			70 (50–81)			50		

Legend: Distribution of discharge diagnoses for patients referred to acute hospital admission by general practitioner (GP) and out-of-hours (OOH) doctor with the ICPC-2 diagnosis: shortness of breath (R01, R02, R03 and R04) in Norway 2017

*IQR* Interquartile range

^a^Percent of referrals with shortness of breath (R01, R02, R03 and R04) and the current ICD-10 discharge diagnosis which are referred by OOH doctor

^b^Percent of women in referrals with shortness of breath (R01, R02, R03 and R04) and the current ICD-10 discharge diagnosis

^c^Relative risk for the different discharge ICD-10 diagnoses for OOH referrals relative to GP referrals, for a 10-years increase in age, and for female patients relative to male patients *compared with all discharge diagnoses after shortness of breath admission*

^d^Relative risk for a 10-years increase in age is not estimated due to no variation in age

### Referrals for abdominal pain

The median patient age for the abdominal pain symptom group referrals was 46 years (Table 3), and 60% were women. Every fourth patient referred with abdominal pain was discharged with a similar symptom diagnosis from ICD-10, abdominal and pelvic pain (R10). The second and third most frequent discharge diagnoses were acute appendicitis (K35) (12%) and cholelithiasis (K80) (6%).

For the abdominal pain symptom group, there was a higher relative risk for the discharge diagnosis to be ileus (K56) if the patient was referred from an OOH doctor compared with a referral from a GP (RR = 2.12 [95%CI: 1.84–2.45]). The opposite was found for diverticular disease (K57) with a lower relative risk of being discharged with diverticular disease if referred from OOH compared to GP (RR = 0.51 [95%CI: 0.46–0.56]). The relative risk for the discharge diagnosis of abdominal and pelvic pain (R10) was higher for women compared to men (RR = 1.38 [95%CI: 1.32–1.44]), but lower for acute appendicitis (K35) (RR = 0.59 [95%CI: 0.55–0.62]), acute pancreatitis (K85) (RR = 0.54 [95%CI: 0.47–0.61]) and calculus of kidney and ureter (N20) (RR = 0.40 [95%CI: 0.34–0.47]).

### Referrals for chest pain

The median patient age in the chest pain symptom group was 62 years, and 44% were women (Table 4). One third of patients referred with chest pain were discharged with the ICD-10 code describing the similar symptom, pain in throat and chest (R07). AMI (I21) accounted for 12%, and angina pectoris (I20) for 10%.

Among patients in the chest pain symptom group, a discharge diagnosis of heart failure (I50) was associated with higher age (RR = 2.13 [95%CI: 1.93–2.36]). Women referred with chest pain had a lower relative risk of being discharged with a diagnosis related to ischemic heart disease: AMI (I21) (RR = 0.54 [95% CI 0.50–0.59]), angina pectoris (I20) (RR = 0.70 [95%CI: 0.64–0.77]) and chronic ischemic heart disease (I25) (RR = 0.47 [95%CI 0.40–0.55]) compared with men.

### Referrals for shortness of breath

In the shortness of breath symptom group the median age was 70 years, and 50% were women (Table 5). Heart failure (I50), pneumonia (J12–18) and chronic obstructive pulmonary disease (J44) were the most common discharge diagnoses with 12, 11 and 8%, respectively. The discharge diagnoses after referral for shortness of breath showed a larger variation compared to abdominal pain and chest pain and had fewer discharges with a symptom describing diagnosis. Among patients discharged with the diagnosis acute bronchiolitis (J21) 75% were less than 2 years.

### Disease specific referral diagnoses: pneumonia, appendicitis, AMI, and stroke

The four referral diagnoses: pneumonia (R81), appendicitis (D88), AMI (K75), and stroke (K90) accounted for 16,811 admissions (6% of all). 59% of the patients referred with the diagnose pneumonia (R81) were discharged with a corresponding ICD-10 pneumonia-diagnosis (J12–18) (Table 6). Only 1% of patients referred with pneumonia were discharged with pulmonary embolism (I26), and 0,5% with AMI (I21).

**Table 6:** Discharge diagnoses for patients referred to acute hospital admission with the diagnosis pneumonia (R81). Relative risk for different discharge ICD-10 diagnoses by explanatory variables (referrals, age and gender), compared with discharged with pneumonia (J12–18)

Discharge ICD-10 diagnoses	All		OOH referrals			Age			Gender (F)		
	N	%	%^a^	RR^c^	95% CI	Median (IQR)	RR^c^	95% CI	%^b^	RR^c^	95% CI
Pneumonia (J12–18)	5173	59	52			72 (57–83)			51		
COPD (J44)	488	6	56	1.09	0.92–1.29	75 (69–82)	1.21	1.15–1.27	52	1.06	0.90–1.26
Influenza (J10)	328	4	59	1.27	1.02–1.57	76 (65–85)	1.14	1.08–1.21	51	1.01	0.82–1.24
Other diagnoses chapter J (respiratory)	1041	12	51	0.98	0.88–1.10	69 (44–80)	0.90	0.88–0.91	49	0.93	0.84–1.04
Diagnoses chapter I (circulatory)	420	5	49	0.84	0.70–1.01	81 (71–88)	1.32	1.24–1.41	50	0.96	0.80–1.15
Diagnoses chapter N (genitourinary)	285	3	57	1.14	0.91–1.44	81 (71–86)	1.26	1.18–1.35	47	0.86	0.69–1.08
Other	1058	12	50	0.91	0.82–1.02	73 (64–83)	1.07	1.04–1.10	45	0.82	0.73–0.91
All	8793	100	52			73 (60–83)			50		

Legend: Distribution of discharge diagnoses for patients referred to acute hospital admission by general practitioner (GP) and out-of-hours (OOH) doctor with the ICPC-2 referral code pneumonia (R81) in Norway 2017

*IQR* Interquartile range

^a^Percent of OOH doctor in referrals with pneumonia (R81) diagnosis and the current ICD-10 discharge diagnosis

^b^Percent of women in referrals with pneumonia (R81) diagnosis and the current ICD-10 discharge diagnosis

^c^Relative risk for the different ICD-10 discharge diagnoses for OOH referrals relative to GP referrals, for a 10-years increase in age, and for female patients relative to male patients *compared with discharge with* *pneumonia (J12–18)*

Of patients referred with appendicitis (D88) 51% were discharged with the corresponding ICD-10 diagnosis acute appendicitis (K35), and 24% with the symptom describing diagnosis abdominal and pelvic pain (R10) (Table 7).

**Table 7:** Discharge diagnoses for patients referred to acute hospital admission with the diagnosis appendicitis (D88). Relative risk for different discharge ICD-10 diagnoses by explanatory variables (referrals, age and gender), compared with discharged with acute appendicitis (K35)

Discharge ICD-10 diagnoses	All		OOH referrals			Age			Gender (F)		
	N	%	%^a^	RR^c^	95% CI	Median (IQR)	RR^c^	95% CI	%^b^	RR^c^	95% CI
Acute appendicitis (K35)	1318	51	62			29 (19–44)			43		
Abdominal and pelvic pain (R10)	613	24	61	0.95	0.84–1.07	22 (16–32)	0.82	0.79–0.86	68	2.04	1.77–2.35
Diverticular disease (K57)^d^	79	3	41	0.68	0.44–1.07	46 (29–56)	1.71	1.58–1.85	48	0.93	0.61–1.40
Other diagnoses chapter K (digestive)	180	7	59	0.94	0.71–1.24	29 (19–49)	1.06	0.98–1.14	48	1.20	0.91–1.57
Diagnoses chapter N (genitourinary)	172	7	70	1.32	0.98–1.79	29 (19–41)	0.94	0.86–1.01	87	7.13	4.65–10.94
Other	219	8	54	0.72	0.57–0.93	24 (13–38)	0.86	0.79–0.93	50	1.32	1.03–1.69
All	2581	100	61			27 (18–82)			53		

Legend: Distribution of discharge diagnoses for patients referred to acute hospital admission by general practitioner (GP) and out-of-hours (OOH) doctor with the ICPC-2 referral code appendicitis (D88) in Norway 2017

*IQR* Interquartile range

^a^Percent of OOH doctor in referrals with appendicitis (D88) diagnosis and the current ICD-10 discharge diagnosis

^b^Percent of women in referrals with appendicitis (D88) diagnosis and the current ICD-10 discharge diagnosis

^c^Relative risk for the different ICD-10 discharge diagnoses for OOH referrals relative to GP referrals, for a 10-years increase in age, and for female patients relative to male patients *compared with discharge with acute appendicitis (K35)*

^d^Poisson regression was used to estimate RR

For patients referred with the diagnosis AMI (K75), 43% were discharged with the corresponding ICD-10 diagnosis AMI (I21), 12% with pain in throat and chest (R07), and 7% with angina pectoris (I29) (Table 8). The discharge diagnosis heart failure (I50) was associated with higher age (RR = 1.96 [95%CI: 1.51–2.55]).

**Table 8:** Discharge diagnoses for patients referred to acute hospital admission with the diagnosis acute myocardial infarction (K75). Relative risk for different discharge ICD-10 diagnoses by explanatory variables (referrals, age and gender), compared with discharged with acute myocardial infarction (I21)

Discharge ICD-10 diagnoses	All		OOH referrals			Age			Gender (F)		
	N	%	%^a^	RR^c^	95% CI	Median (IQR)	RR^c^	95% CI	%^b^	RR^c^	95% CI
Acute myocardial infarction (I21)	583	43	63			66 (56–76)			25		
Pain in throat and chest (R07)^d^	160	12	54	0.71	0.54–0.93	62 (53–74)	0.79	0.71–0.87	36	1.81	1.35–2.42
Angina pectoris (I20)	93	7	40	0.47	0.32–0.69	72 (64–81)	1.21	1.04–1.41	27	0.87	0.56–1.34
Chronic ischaemic heart disease (I25)	60	4	47	0.47	0.29–0.77	61 (54–67)	0.74	0.61–0.90	15	0.68	0.34–1.37
Atrial fibrillation and flutter (I48)	49	4	51	0.73	0.43–1.23	76 (69–82)	1.51	1.21–1.89	43	1.37	0.78–2.41
Heart failure (I50)	41	3	66	1.30	0.71–2.35	81 (73–88)	1.96	1.51–2.55	51	1.50	0.81–2.75
Other diagnoses chapter I (circulatory)	121	9	62	0.99	0.71–1.38	71 (57–81)	1.05	0.93–1.20	37	1.48	1.04–2.11
Other diagnoses chapter R (symptoms)	63	5	60	0.95	0.59–1.52	71 (60–82)	1.09	0.90–1.31	43	1.85	1.12–3.06
Diagnoses chapter J (respiratory)	62	5	55	0.83	0.52–1.32	77 (69–83)	1.52	1.25–1.85	39	1.16	0.70–1.91
Other	127	9	42	0.52	0.38–0.72	70 (59–83)	1.03	0.91–1.16	46	1.94	1.40–2.68
All	1359	100	57			68 (57–79)			32		

Legend: Distribution of discharge diagnoses for patients referred to acute hospital admission by general practitioner (GP) and out-of-hours (OOH) doctor with the ICPC-2 referral code acute myocardial infarction (K75) in Norway 2017

*IQR* Interquartile range

^a^Percent of OOH doctor in referrals with myocardial infarction (K75) diagnosis and the current ICD-10 discharge diagnosis

^b^Percent of women in referrals with myocardial infarction (K75) diagnosis and the current ICD-10 discharge diagnosis

^c^Relative risk for the different ICD-10 discharge diagnoses for OOH referrals relative to GP referrals, for a 10-years increase in age, and for female patients relative to male patients *compared with discharge with* *acute myocardial infarction (I21)*

^d^Poisson regression was used to estimate RR

After referral with the diagnosis stroke (K90), 30% of the patients were discharged with the ICD-10 diagnosis cerebral infarction (I63), 10% with transient cerebral ischemic attack (TIA) (G45) and 4% with intracerebral haemorrhage (I61), whereas 56% had other diagnoses (Table 9).

**Table 9:** Discharge diagnoses for patients referred to acute hospital admission with the diagnosis stroke (K90). Relative risk for different discharge ICD-10 diagnoses by explanatory variables (referrals, age and gender), compared with discharged with cerebral infarction (I63)

Discharge ICD-10 diagnoses	All		OOH referrals			Age			Gender (F)		
	N	%	%^a^	RR^c^	95% CI	Median (IQR)	RR^c^	95% CI	%^b^	RR^c^	95% CI
Cerebral infarction (I63)	1243	30	58			75 (67–83)			42		
TIA (G45)	398	10	60	1.07	0.90–1.27	76 (67–84)	1.02	0.95–1.09	53	1.38	1.16–1.64
Intracerebral haemorrhage (I61)	145	4	63	1.19	0.86–1.63	77 (67–83)	0.99	0.87–1.11	49	1.28	0.93–1.75
Diagnoses chapter R (symptoms)	589	14	58	0.95	0.83–1.07	69 (53–80)	0.82	0.80–0.83	51	1.29	1.14–1.46
Other diagnoses chapter I (circulatory)	382	9	57	0.95	0.80–1.13	74 (65–83)	0.96	0.89–1.02	43	1.05	0.87–1.25
Other diagnoses chapter G (nervous)	366	9	60	0.95	0.80–1.14	67 (51–77)	0.76	0.74–0.79	52	1.36	1.15–1.60
Diagnoses chapter J (respiratory)	133	3	48	0.69	0.50–0.95	81 (73–86)	1.36	1.17–1.58	40	0.79	0.56–1.10
Other^d^	822	20	54	0.90	0.81–1.00	74 (63–82)	0.91	0.87–0.94	50	1.25	1.13–1.39
All	4078	100	57			74 (63–83)			47		

Legend: Distribution of discharge diagnoses for patients referred to acute hospital admission by general practitioner (GP) and out-of-hours (OOH) doctor with the ICPC-2 referral code stroke (K90) in Norway 2017

*IQR* Interquartile range

^a^Percent of OOH doctor in referrals with stroke (D90) diagnosis and the current ICD-10 discharge diagnosis

^b^Percent of women in referrals with stroke (D90) diagnosis and the current ICD-10 discharge diagnosis

^c^Relative risk for the different ICD-10 discharge diagnoses for OOH referrals relative to GP referrals, for a 10-years increase in age, for female patients relative to male patients *compared with discharge with acute cerebral infarction (I63)*

^d^Poisson regression was used to estimate RR

## Discussion

### Main results

Of all GP contacts only 1% resulted in a referral for acute admission to somatic hospital, whereas OOH doctors referred 11%. Referral rates for GP and OOH contacts vary greatly by referral diagnosis. Abdominal pain, chest pain and shortness of breath were the dominant symptom diagnoses in referrals and had a considerable variation in discharge diagnoses. For both abdominal pain and chest pain the corresponding symptom diagnosis was the most frequent. For the referral diagnoses pneumonia, appendicitis, AMI and stroke the corresponding discharge diagnoses were dominant. Some of the discharge diagnoses were associated with high patient age, female gender, and if the referral came either from a GP or an OOH doctor.

### Strengths and limitations

The study includes all residents in Norway in 2017, all GP and OOH doctor contacts and all acute referrals to somatic hospitals. Therefore, there is no selection bias. The data sources are registries containing activity data delivered to manage funding of primary and specialist healthcare, and therefore presumably accurate and complete. This ensures the representativity for the situation in Norway.

In our analyses we used only the primary diagnosis. Therefore, we may have missed some information on the reason for referral in cases with more than one diagnosis given.

Diagnosis coding may vary between doctors, both in general practice and in OOH services. In a clinical situation with abdominal pain when appendicitis is suspected, the physician may apply either a symptom diagnosis *abdominal pain/cramps general (D01)* or the disease diagnose *appendicitis (D88)*. Probably, in some cases a more precise or severe diagnosis than the right one according to diagnosis criteria may be used to prepare the hospital receiving the patient. Distinction between symptom and disease diagnosis in referrals must be interpreted with caution. In a prehospital setting in helicopter medical services in Finland, ICPC-2 coding was tested for inter-rater reliability for six written fictional cases [23]. The researchers found an overall agreement of only 52%. Nevertheless, research in Norwegian general practice concludes that GPs’ use of ICPC-2 codes corresponds well with the patient records in 85% of the consultations [24]. This supports the use of ICPC-2 diagnosis to describe the reason for referral. Our results reveal a coherence between referral ICPC-2 diagnosis and the hospital discharge diagnosis, supporting the design.

The linkage between the GP and OOH doctor contacts in KUHR and hospital admission in NPR has some uncertainties. The primary care contact prior to admission might be random, and not related to the admission. In a previous study we found a distinct accumulation of GP and OOH contacts within 24 h before acute hospital admission [9, 10]. This indicates that the assumption that a contact within 24 h before an admission is the referral contact, is valid. The present study supports this further by demonstrating the relationship between the referral and the discharge diagnoses.

### Referral rates and reasons for referral

Our overall OOH referral rate (11%) is higher than the rate described in studies from England (7 and 10%) [25, 26], and Denmark (4–8%) [27], but lower than a study from a single OOH primary care centre in Norway (14%) [5].

Diagnoses from the ICPC-2 chapter general and unspecified conditions (A) was most frequent for referrals from OOH doctors, similar to findings from other studies [11, 27]. The variation of referral rates for different ICPC-2 diagnoses reflects the severity of the conditions as well as the nature of the services. For atrial fibrillation, the GPs’ referral rate was only 0.01 whereas the OOH referral rate was 0.45. According to national guidelines, newly discovered atrial fibrillation should be referred for immediate further investigations and treatment, whereas chronic atrial fibrillation requires comprehensive GP follow up, but not admission. There is a similar effect for GP contacts with a diagnosis of appendicitis (D88) where as much as 70% were not referred. This probably represent GP follow up, rather than acute assessment. These figures underline the different nature of GP daytime services and OOH services, and therefore the proportions of contacts for a diagnosis related to admission are not directly comparable.

Abdominal pain is a common symptom in general practice, and the second most frequent reason for contacting OOH services [13, 21, 28]. In a Norwegian study of patients offered acute appointment with a GP because of abdominal pain, the GP referred 26% acutely to hospital [13]. In our study only 4% of GP patients with abdominal pain/cramps or localized abdominal pain were acutely referred to hospital, illustrating that GPs provide care for both acute and chronic complaints. OOH services provide emergency primary healthcare with only acute appointments and correspond better with the patients in the other Norwegian GP study. For OOH patients with abdominal pain/cramps or localized abdominal pain, 26 and 24% were referred in our material, which fits well with the GP study.

We found that the referral rates for chest pain and heart pain from OOH doctors were 0.37 and 0.66, respectively. In a prospective study from Norwegian OOH services 50 out of 100 patients presenting at the casualty clinic with chest pain as the main symptom were referred to hospital [12]. However, we do not know the referral diagnosis for these referrals.

### Abdominal pain, chest pain and shortness of breath symptom groups

For referrals with abdominal and chest pain symptoms, the most frequent ICD-10 discharge diagnoses were the corresponding symptom-based diagnoses abdominal and pelvic pain (R10) and pain in throat and chest (R07). Our previous study showed that these discharge diagnoses were the overall second and third most frequent discharge diagnoses after acute admissions to hospital, irrespective of referral agent [9, 10]. Such extensive use of symptom diagnoses both in referrals and discharges could serve as a reminder of the diagnostic challenges in both primary care and in hospitals but might as well be an indication of defensive medicine.

Only 1% of patients referred with chest pain were discharged with gastro-oesophageal reflux disease (K01). This could be due to effective gatekeeping treating these patients in primary care and is in line with previous research from Norway where 5 out of 100 patients attending OOH services with chest pain were diagnosed with dyspepsia by OOH doctor, and none were referred to hospital [12].

Chest pain is a core presenting symptom for AMI for both women and men, although atypical symptom presentations in women have received increased attention [15, 29, 30]. We found that women referred with a chest pain diagnosis had a lower relative risk of being discharged with a discharge diagnosis of ischemic heart disease compared with men. This adds to previous knowledge of gender differences in acute ischemic heart disease presentation.

### Referrals for pneumonia, appendicitis, AMI, or stroke

GPs accounted for a somewhat larger share of the disease-specific referral diagnoses pneumonia (R81), appendicitis (D88), AMI (K75), and stroke (K90) than the symptom groups abdominal pain, chest pain, and shortness of breath, suggesting that GPs are more likely than OOH doctors to use specific disease diagnoses. This could be due to better knowledge of the patient’s history or better diagnostic facilities.

For referrals with diagnoses pneumonia, appendicitis and AMI the most frequent discharge diagnoses were the corresponding disease diagnosis. Correspondingly, referrals with the diagnosis stroke were often discharged with a diagnosis of cerebral infarction, TIA, or intracerebral haemorrhage. This indicates that when the referral diagnosis is a disease-specific diagnosis, the primary care doctor is more certain of a specific disease compared with cases where a symptom diagnosis is used. Hospital doctors could take this into consideration when receiving patients referred acutely from primary care with a disease-specific diagnosis.

Patients from OOH services with the referral diagnosis AMI are less likely to be discharged with the diagnosis angina pectoris or chronic ischemic heart disease than patients from general practice. Angina pectoris and chronic ischemic heart disease typically have a less acute presentation, leading to contact with the GP rather than the OOH services, again illustrating that GPs treat cases with a lower degree of urgency.

An adequate gatekeeper role for acute hospital admissions must balance the task to discover all patients with serious conditions with the risk of unnecessary admissions [5, 31]. The aim is to avoid missed diagnosis (false negatives) at an acceptable level of false positives where no or minor disease are revealed in hospital. Low risk taking would lead to defensive medicine, increasing costs without gained health and overdiagnosis. On the other hand, too restrictive referral practice would lead to an increased number of undiagnosed severe conditions. Such underdiagnosis will severely affect the individual patient’s health. Finding the right balance in referral practice is a major challenge for primary care doctors performing a gatekeeper function in prehospital acute care. Continuity of care in general practice may help in this task. Length of patient continuity with a named regular GP is associated with lower use of OOH services, fewer hospital admissions and even lower mortality [32].

Our study reveals that one third of patients referred with chest pain and one quarter of patients referred with abdominal pain were discharged with a symptom diagnosis, hence no severe condition was revealed at hospital. Likewise, 24% of patients referred with appendicitis, and 12% referred with AMI were discharged with a symptom describing discharge diagnosis. We believe this to be a reasonable level of accuracy, but attention to this topic should be high and an objective for further research. The topic of referral practice should be emphasized in medical education, and policy makers should be aware of the issue over- and underdiagnosis.

### Lessons learned

Referrals to hospital are always a matter of clinical medical assessment. In a gatekeeping system this also applies for emergencies. Both GPs and OOH doctors seem to perform gatekeeping for acute hospital admissions based on their setting and the different patient populations. Our findings suggest that there should be an accept of more symptom-based referrals from OOH services. The GP’s knowledge of the patient’s medical history is valuable also when performing gatekeeping for acute referrals to hospital. The large variation of referral diagnoses implies that a broad medical competence is necessary when assessing emergencies.

## Conclusions

Referral rates for OOH contacts were much higher than for GPs’ contacts, and showed considerable variation by different diagnoses, thus reflecting the severity of the conditions and the nature of the services. Abdominal pain and chest pain were two major reasons for referral, and the most frequent discharge diagnosis for both was the corresponding ICD-10 symptom describing diagnosis. Women referred with chest pain were less likely to be discharged with a diagnosis reflecting ischemic heart disease, whereas women referred with AMI were more often discharged with the diagnosis pain in throat and chest.

## Data Availability

The data used in this study are delivered from The Norwegian Directorate of Health and Statistics Norway, with restrictions only to be used under licence for researchers in the current study, and so are not publicly available. However, the registry data used in this study will be available from the authors upon reasonable request and with included permission from The Norwegian Directorate of Health, Statistics Norway, the Regional Ethical Committee, and Norwegian Data Protection Authority.

## Abbreviations

GP: General practitioner
OOH: Out-of-hours
AMI: Acute myocardial infarction
ICPC-2: International Classification of Primary Care, second edition
RGP: Regular general practitioner
KUHR: Control and Payment of Reimbursement to Health Service Providers database
HELFO: Norwegian Health Economics Administration
NPR: Norwegian Patient Registry
ICD-10: The International Statistical Classification of Diseases and Related Health Problems version 10
SSB: Statistics Norway
ED: Emergency department
GLM: Generalized linear models
CI: Confidence intervals
IQR: Interquartile range
TIA: Transient cerebral ischemic attack
